# Soluble apoE/Aβ complex: mechanism and therapeutic target for *APOE4*-induced AD risk

**DOI:** 10.1186/1750-1326-9-2

**Published:** 2014-01-04

**Authors:** Leon M Tai, Shipra Mehra, Varsha Shete, Steve Estus, G William Rebeck, Guojun Bu, Mary Jo LaDu

**Affiliations:** 1 Department of Anatomy and Cell Biology, University of Illinois at Chicago, 808 S. Wood St., M/C 512, Chicago, IL 60612, USA; 2 Sanders-Brown Center on Aging, University of Kentucky, Lexington, KY 40536, USA; 3 Department of Neuroscience, Georgetown University, Washington, DC 20057, USA; 4 Department of Neuroscience, Mayo Clinic, Jacksonville, FL 32224, USA

**Keywords:** Alzheimer’s disease, Amyloid beta, Apolipoprotein E, Apolipoprotein E/amyloid beta complex, Oligomeric amyloid beta, Lipoprotein, Lipidation

## Abstract

The *APOE4* allele of apolipoprotein E (apoE) is the greatest genetic risk factor for Alzheimer’s disease (AD) compared to *APOE2* and *APOE3*. Amyloid-β (Aβ), particularly in a soluble oligomeric form (oAβ), is considered a proximal cause of neurodegeneration in AD. Emerging data indicate that levels of soluble oAβ are increased with *APOE4*, providing a potential mechanism of *APOE4*-induced AD risk. However, the pathway(s) by which apoE4 may increase oAβ levels are unclear and the subject of continued inquiry. In this editorial review, we present the hypothesis that apoE isoform-specific interactions with Aβ, namely apoE/Aβ complex, modulate Aβ levels. Specifically, we propose that compared to apoE3, apoE4-containing lipoproteins are less lipidated, leading to less stable apoE4/Aβ complexes, resulting in reduced apoE4/Aβ levels and increased accumulation, particularly of oAβ. Evidence that support or counter this argument, as well as the therapeutic significance of this pathway to neurodegeneration, are discussed.

## Introduction

Sporadic Alzheimer’s disease (AD) accounts for more than 95% of all AD cases and the *APOE4* allele of apolipoprotein E (apoE) is the greatest genetic risk factor; two copies of the *APOE4* allele increase AD risk up to 15-fold relative to *APOE3. APOE4* carriers account for more than half of AD patients and *APOE4* accelerates onset of cognitive impairment by 7-9 years per allele [1,2]. The proposed mechanism(s) through which *APOE4* increases AD risk are multifactorial, including both amyloid-β (Aβ)-dependent effects, i.e. modulation of Aβ levels, aggregation, neurotoxicity and neuroinflammation, and Aβ-independent effects, i.e. neuronal development, glucose metabolism, brain activity and lipid metabolism (reviewed in [3]). In this editorial review, we propose the hypothesis that apoE isoform-specific formation of soluble apoE/Aβ complex modulates levels of neurotoxic Aβ, providing a basis for *APOE4*-induced AD risk. Therapeutic implications are presented, as well as arguments counter to this hypothesis.

N.B.: For the purpose of this paper, Aβ species will be identified with as much detail as allowed by the detection method of a specific publication i.e. "soluble Aβ42" if measured in a soluble extraction fraction or soluble sample with an Aβ42-specific ELISA or "soluble oAβ" if measured in a similar sample using an oligomer-specific detection method (for example, [4]). More general classes of Aβ species will be referred to simply as Aβ or soluble Aβ.

## Does *APOE* modulate soluble Aβ levels?

Genetic and experimental evidence posits soluble Aβ as the proximal neurotoxin in AD. However, as a number of potentially neurotoxic forms of the Aβ peptide exist, the identity of the exact neurotoxic form of the peptide, if there is indeed only one, is unclear. The different forms of soluble Aβ have ambiguous and often overlapping definitions based on the method of detection (e.g. biochemical or immunohistochemical analysis) and include; soluble Aβ [5-7], oligomeric Aβ (oAβ [8-11]) and Aβ present in amyloid plaques [12-16]. Further complications include; 1) a dynamic compartmentalization between the different types of Aβ in the CNS i.e. between plaques and soluble Aβ [17,18]; and 2) different forms of Aβ contributing to neurodegeneration at different stages of the disease [19]. While plaque burden may not specifically correlate with cognitive dysfunction in AD, an emerging consensus is that soluble Aβ42 and oAβ represent major proximal, neurotoxic species in AD [15,20]. Indeed, soluble Aβ and oAβ correlate with cognitive decline and disease severity in humans [21], and oAβ levels are associated with memory decline in transgenic mice expressing familial-AD (FAD) mutations (FAD-Tg) (for review [21]).

In both FAD-Tg mice and humans, *APOE4* is associated with higher levels of insoluble Aβ, the result of an increase in total plaque burden and extracellular Aβ compared to *APOE2* and *APOE3*[22-25]. Importantly, in FAD-Tg mice crossed with apoE-targeted replacement mice (apoE-TR), there are higher levels of both soluble Aβ42 [12,23] and soluble oAβ (EFAD mice) [12], with *APOE4* compared to *APOE3*. In addition, using a gene transfer approach, viral expression of *APOE4* increases, and *APOE2* decreases, ISF Aβ42 levels in FAD-Tg mice [18]. The increased soluble Aβ levels in FAD-Tg mice expressing *APOE4* have been confirmed in humans [4,26,27]. In AD patients with *APOE4* compared to *APOE3,* oAβ levels are higher surrounding amyloid plaques [26], in isolated synaptoneurosomes [26], synaptosome enriched extracts [28], and in TBS brain extracts [27]*.* Furthermore, in human CSF, oAβ levels are increased in AD patients compared to non-AD (NAD) and are greater in *APOE4/4* AD patients compared to *APOE3/3* AD patients [4].

### Interpretation

As soluble Aβ is considered a major neurotoxin in AD, the apparent correlation between *APOE4* and increased levels of particularly soluble oAβ suggests that understanding the underlying pathway(s) that mediates this effect may be critical to understanding the *APOE4*-induced risk for AD. In turn, this fundamental biology could inform rational drug design and development of successful AD therapeutics.

## Does *APOE* modulate soluble apoE/Aβ levels and stability?

### Historical perspective

ApoE isoform-specific effects on apoE/Aβ complex levels may mediate the increased soluble Aβ and oAβ levels that correlate with *APOE4*. Research efforts to determine the effect of apoE isoform on exogenous complex formation (Table 1) or endogenous apoE/Aβ complex levels (Table 2) have been ongoing for the last ~20 years (see Table 1). These studies demonstrate that the method of detection and the source of the components for apoE/Aβ complex are critical parameters for experimental outcomes.

**Table 1:** Effect of apoE isoform on soluble exogenous apoE/Aβ complex levels

**Study**	**Human apoE source**	**Aβ source**	**apoE:Aβ Molar Ratio**	**Detection Method**	**Results**
Strittmatter *et al*, *1993*[29,30]	Human CSF (NAD & AD)	Syn. Aβ40, 1-28, 12-28	* ^(100μl CSF:2.5mM)^	SDS-PAGE (Reducing), WB	apoE binds to Aβ40, 1-28, 12-28
	Human plasma (Purified)	Syn. Aβ40	1:170	SDS-PAGE (Non-reducing), WB	apoE4/Aβ > apoE3/Aβ Stability at 4.6 pH = apoE3/Aβ > apoE4/Aβ ^(<10% apoE binds Aβ)^
Wisniewski *et al*, 1993 [31]	Human CSF (NAD & AD)	Syn. Aβ40, Aβ42	* ^(50μg CSF:2μg/ml)^	SDS-PAGE (Reducing), WB	apoE/Aβ at 34kDa
Sanan *et al* 1994 [32]	Human plasma (Purified)	Syn. Aβ28	1:139	SDS-PAGE (Non-reducing), WB	apoE3/Aβ > apoE4/Aβ
LaDu *et al*, 1994 [33]	HEK293 (CM)	Syn. Aβ40	1:357		apoE3/Aβ > apoE4/Aβ
LaDu *et al*, 1995 [34]	HEK293 (Purified & CM), Human plasma (Native & purified)	Syn. Aβ40	1:357	SDS-PAGE (Non-reducing), WB	CM & plasma (native): apoE3/Aβ40 > apoE4/Aβ40 Purified apoE (both sources): apoE3/Aβ40 = apoE4/Aβ40
Castano *et al*, 1995 [35]	Recombinant ^#^	Syn. Aβ40	1:169	SDS-PAGE (Non-reducing), WB	apoE3/Aβ40 at 40kDa
Naslund *et al*, 1995 [36]	Recombinant ^#^	Syn. Aβ40, Aβ42	1:136	SDS-PAGE (Non-reducing & reducing), WB	Non-reducing: apoE3/Aβ = apoE4/Aβ Reducing: higher molecular mass complexes
Golabek *et al*, 1995 [37]	Recombinant ^#^	Syn. Aβ40	1:8.5	SDS-PAGE (Non-reducing), WB	apoE/Aβ at >36kDa
Golabek *et al*, 1996 [38]	Recombinant ^#^	Syn. Aβ40	* ^(0-150nM:2.5pmol)^	Solid plate assay	apoE2/Aβ = apoE3/Aβ = apoE4/Aβ
Shuvaev & Siest *et al*, 1996 [39]	Human plasma (Purified)	Syn. Aβ40	1:130	Surface plasmon resonance	apoE3/Aβ > apoE4/Aβ = apoE2/Aβ^(↑ apoE3/Aβ with ↑ salt concentration^ & ^unaffected in pH 6-8)^
Chan *et al*, 1996 [40]	*Ecoli* (Purified), Human plasma (Purified)	Syn. Aβ40	1:3-1:11	SDS-PAGE (Non-reducing), WB, Gel filtration	apoE4/Aβ = apoE3/Aβ = apoE2/Aβ^(Both sources gave same results, Aβ^ & ^apoE tetramer co-migrate)^
Zhou *et al*, 1996 [41]	RAW264 (CM)	Syn. Aβ40	1:170	SDS-PAGE (Non-reducing), WB	apoE3/Aβ >> apoE4/Aβ (ND)
LaDu *et al*, 1997 [42]	HEK293 (CM), Human plasma (Native & Purified), rat & rabbit apoE (native)	Syn. Aβ40	1:357 (CM), 1:715 (Plasma)	SDS-PAGE (Non-reducing), WB	Native: apoE2/Aβ = apoE3/Aβ = rabbit apoE/Aβ > apoE4/Aβ (ND) = rat apoE/Aβ Purified: apoE2/Aβ = apoE3/Aβ > apoE4/Aβ (ND) ^(Both CM & human plasma - native gave same results)^
Yang *et al*, 1997 [43]	CHO (CM), Human plasma	Syn. Aβ40	1:97 (CM), 1:850 (Plasma)	SDS-PAGE (Non-reducing), WB	apoE3/Aβ = apoE2/Aβ >> apoE4/Aβ (ND) ^(Both sources gave same results)^
Aleshkov *et al*, 1997 [44]	BHK21 (CM), Recombinant ^#^ (Lipidated), Human plasma	Syn. Aβ40	1:126	SDS-PAGE (Non-reducing), WB	Recombinant & CM (apoE monomer): apoE2/Aβ > apoE3/Aβ >> apoE4/Aβ Plasma: apoE3/Aβ > apoE4/Aβ
Pillot *et al*, 1997 [45]	Recombinant ^#^	Syn. Aβ29-40/42	1:5-1:100	SDS-PAGE (Non-reducing), WB	apoE2/Aβ = apoE3/Aβ > apoE4/Aβ (ND) ^(Dose dependent ↑ complex with ↑ ratio Aβ:apoE3 or apoE2)^
Russo *et al*, 1998 [46]	Human plasma apoE ^#^	Syn. Aβ42, Human Aβ (Brain)	* ^(17.7pmol:100μM)^	IP with SDS-PAGE ^(Non-Reducing?),^ WB	apoE/Aβ complex at 40kDa
Pillot *et al*, 1999 [47]	Recombinant ^#^	Syn. Aβ29-40, Aβ29-42	1:50	SDS-PAGE (Non-reducing), WB	CTF-apoE/Aβ > NTF-apoE/Aβ (ND)
Yamauchi *et al*, 1999 [48]	Recombinant ^#^ (Non-lipidated & lipidated)	Syn. Aβ42	1:2-1250	ELISA	apoE2/Aβ > apoE3/Aβ > apoE4/Aβ^(No differences in lipidated vs. non-lipidated in apoE isoform)^
Aleshkov *et al*, 1999 [49]	BHK1 (CM)	Syn. Aβ40	1:125	SDS-PAGE (Non-reducing), WB	apoE2/Aβ = apoE2-Thr194-Ala/Aβ = apoE4-Arg158-cys/Aβ
Golabek *et al*, 2000 [50]	Recombinant ^#^ Human plasma (Purified)	Syn. Aβ40	1:8.5	SDS-PAGE (Non-reducing), WB	Recombinant: NTF-apoE3/Aβ > CTF-apoE3/Aβ Plasma: apoE/Aβ at 38kDa
Tokuda *et al*, 2000 [51]	RAW264 & HEK293 (CM, delipidated), Sf9 insect cells (Delipidated & lipidated)	Syn. Aβ40, Aβ42	* ^(0-150nM/2.5pmol)^	ELISA	CM & Sf9 (Lipidated): apoE3/Aβ > apoE4/Aβ All sources (Delipidated): apoE3/Aβ = apoE4/Aβ ^(apoE/Aβ: CM > Sf9 lipidated)^
Drouet *et al*, 2001 [52]	*Ecoli* (Purified)	Syn. Aβ29-40	1:100	SDS-PAGE (Non-reducing), WB	apoE/Aβ-CTF: apoE2/Aβ = apoE3/Aβ > apoE4/Aβ (ND)
Zhou *et al*, 2002 [53]	RAW264 (CM), CSF (NAD E3/3, PAD E3/4, AD E4/4)	Syn. Aβ40	1:250 (CM), * (CSF) ^(70μl CSF:100μM)^	co-IP, SDS-PAGE (Non-reducing), WB	CM: apoE3/Aβ >> apoE4/Aβ CSF: *APOE*33/Aβ > *APOE*34/Aβ = *APOE*44/Aβ (ND) ^(With BMEless apoE3/Aβ)^
Bentley *et al*, 2002 [54]	HEK293 (CM)	Syn. Aβ40	1:340	SDS-PAGE (Non-reducing), WB	apoE3/Aβ > apoE4/Aβ = apoE3-Ala-112/Aβ = apoE4-Lys-112/Aβ ^(ApoE3-Thr-61/Aβ = apoE4-Thr-61/Aβ = no complex)^
Gylys KH *et al*, 2003 [55]	Recombinant ^#^ (Lipidated)	Syn. Aβ40	1:5.6	SDS-PAGE (Non-reducing), WB	apoE3/sAβ > apoE3/agg Aβ
Manelli *et al*, 2004 [56]	HEK293 (CM)	Syn. Aβ42	1:33	SDS-PAGE (Non–reducing), WB	apoE3/oAβ > apoE3/Aβ fibrils > apoE4/oAβ > apoE4/Aβ fibrils
Phu *et al*, 2005 [57]	Recombinant ^#^	Syn. AEDANS-F4C- Aβ42	1:1	FRET	Soluble complex: CTF-apoE/Aβ
Stratman *et al*, 2005 [58]	Recombinant ^#^ (Lipidated)	Syn. Aβ40	1:500	ELISA	Intermediate agg Aβ40: apoE4/Aβ >> apoE2/Aβ = apoE3/Aβ
Morikawa *et al*, 2005 [59]	Immortalized astrocytes apoE-TR (CM immuno-purified), Primary astrocytes GFAP-apoE-Tg (CM Immuno-purified)	Syn. Aβ40	1:4.5-22.5	SDS-PAGE (Reducing & non-reducing), WB ^(Physiological buffer in non - reducing)^	Reducing: apoE3/Aβ > apoE4/Aβ Non-reducing: apoE3/Aβ = apoE4/Aβ
Wellnitz *et al*, 2005 [60]	N2a (CM)	Syn. Aβ42	1:0.1-1000	SDS-PAGE (Reducing), WB	CTF-apoE/hexameric Aβ: apoE4/Aβ > apoE3/Aβ = apoE2/Aβ
Petrlova *et al*, 2011 [61]	*Ecoli* (Purified & lipidated)	Syn. Aβ40	1:3.3	EPR spectroscopy	Purified apoE: apoE3/oAβ > apoE4/oAβ Lipidated apoE: apoE3/Aβ > apoE4/Aβ ^(CTF apoE bind Aβ)^
Cerf *et al*, 2011 [62]	*Ecoli* (Purified)	Syn. Aβ42	1:25-100	SDS-PAGE (Non-reducing), WB	apoE monomers/agg Aβ: apoE3/Aβ = apoE4/Aβ ^(Stabilize oAβ42: apoE4 > apoE3)^
Hashimoto *et al*, 2012 [27]	Immortalized astrocytes apoE-TR (CM immuno-purified)	Syn. Aβ42	1:0.083	SDS-PAGE (Reducing), WB	No complex measured
					Lipidated apoE stabilizes oAβ42: apoE4 > apoE3 > apoE2
LaDu *et al*, 2012 [63]	Human Plasma (NAD), Rat Astrocyte (CM, isolated, purified delipidated)	Syn. Aβ40	1:5-16 (Plasma); 1:36 (Rat ACM); 1:357 (Rat isolated & purified)	SEC, SDS-PAGE (Non–reducing), WB	Aβ co-elutes with apoE containing lipoproteins: Human plasma (70%) > rat ACM (53%) Monomer apoE/Aβ (45kDa) & dimer apoE/Aβ 97kDa : rat isolated > rat purified
Ly *et al*, 2013 [64]	*Ecoli* (Purified)	Syn. Aβ40	1-4:1, 1:2	Laser fluorescence spectroscopy	Stable complex- apoE3L-Cys-264/oAβ > apoE4-Cys-264/oAβ ^(apoE3L means "apoE3 like" with Cys112-Ser)^
Tai *et al*, 2013 [4]	HEK293 (CM)	Syn. Aβ42	1:0.005-50	ELISA	Total complex: apoE2/Aβ = apoE3/Aβ = apoE4/Aβ SDS stable: apoE2/Aβ > apoE3/Aβ > apoE4/Aβ pH=5: apoE2/Aβ = apoE3/Aβ > apoE4/Aβ
Verghese *et al*, 2013 [65]	Recombinant ^#^ (Lipidated)	APP H4 neuroglioma (CM), Syn. Aβ40/42	1:0.02-0.05 (CM), 1:0.2-1 (Syn.)	Density gradient ultracentrifugation, SEC, ELISA, FCS	Monomeric Aβ free (95-97%) >> apoE3/Aβ = apoE4/Aβ ^(Lipidated apoE poorly binds binds Aβ)^
	Immortalized astrocytes apoE-TR (CM immuno-purified), Primary astrocytes GFAP-apoE-Tg (CM Immuno-purified)	APP H4 neuroglioma (CM), 7PA2 cells (CM)	1:0.04 (H4), 1:0.05 (7PA2)	SEC, ELISA, FCS	Higher order Aβ species (free) >> apoE3/Aβ = apoE4/Aβ
	Human CSF (Pooled non-concentrated, NAD)	APP H4 neuroglioma (CM)	* ^(800μl CSF:50ng/ml)^	SEC	95% in vitro Aβ (free) = in vivo Aβ (free) >> apoE/Aβ

^#^, Commercially purchased recombinant human apoE; *, apoE:Aβ ratio unknown; Lipidated apoE is either with POPC, reconstituted "HDL" or plasma lipoprotein.

ACM, Astrocyte conditioned media; AD, Alzheimer’s disease; agg Aβ, Aggregated Aβ; BME, β - mercaptoethanol; CSF, Cerebrospinal fluid; CM, Conditioned media; co-IP, co - immunoprecipitation; CTF, C-terminal fragment; EPR, Electron paramagnetic resonance; ELISA, Enzyme-linked immunosorbent assay; FCS, Fluorescence correlation spectroscopy; FRET, Fluorescence resonance energy transfer; IP, Immunoprecipitation; NAD, Non-AD or non-dementia control; ND, Not detectable; NTF, N-terminal fragment; oAβ, Oligomeric Aβ; PAD, Probable AD; PAGE, Polyacrylamide gel electrophoresis; sAβ, Soluble Aβ; Sf9, *Spodoptera frugiperda* insect cells; SDS, Sodium dodecyl sulfate; SEC, Size exclusion chromatography; Syn, Synthetic; Tg, Transgenic; TR, Target replacement; WB, western blot.

**Table 2:** **Effect of apoE isoform on soluble exogenous apoE/A**β **complex levels**

**Study**	**Biological source**	**Detection method**	**Results**
Naslund *et al*, 1995 [36]	Human brain (AD & NAD)	SDS-PAGE (Non-reducing), WB	AD > NAD, No apoE isoform differences measured
Russo *et al*, 1998 [46]	Human brain (AD & NAD)	IP, SDS-PAGE ^(Non-reducing?),^ WB	NAD apoE23/Aβ = NAD apoE33/Aβ = NAD apoE34/Aβ >>
			AD apoE33/Aβ > AD apoE44/Aβ
			SDS & protease digestion stability: NAD > AD
Yamauchi *et al*, 1999 [48]	Human CSF & serum (NAD)	SDS-PAGE (Non-reducing), WB	apoE33/Aβ > apoE44/Aβ (ND)
Hashimoto *et al*, 2012 [27]	Human brain (NAD)	SEC, SDS-PAGE (Reducing), WB	No complex measured, HMW Aβ interacts with apoE on HDL particles
LaDu et al, 2012 [63]	Human plasma (NAD)	SEC, SDS-PAGE (Non-reducing), WB	95% Aβ elutes with lipoproteins
	Human CSF (NAD)		100% Aβ associated with apoE containing lipoproteins, apoE monomer/Aβ (45 kDa) & apoE dimer/Aβ (97 kDa) detected
Tai *et al*, 2013 [4]	Hippocampal homogenates (EFAD mice)	ELISA	SDS stable: E2FAD > E3FAD > E4FAD Total complex: E2FAD = E3FAD > E4FAD
	Human cortical synaptosomes (AD & NAD)		Total complex:
			• NAD > AD
			• NAD apoE33/Aβ = NAD apoE4X/Aβ > > AD apoE33/Aβ > AD apoE4X/Aβ
			SDS stable:
			• NAD apoE33/Aβ > > NAD apoE4X/Aβ & AD apoE33/Aβ > AD apoE4X/Aβ
	Human CSF (AD & NAD)		• NAD apoE33/Aβ > > NAD apoE4X/Aβ
Verghese, *et al*, 2013 [65]	Human CSF (NAD)	SEC, ELISA	95% Aβ (free) > > apoE33/Aβ = apoE44/Aβ
			No apoE isoform differences
			^(In co-elution peak stoichiometric ratio of apoE:Aβ = 1:0.0002-0.0003)^

AD, Alzheimer’s disease patients; CSF, Cerebrospinal fluid; ELISA, Enzyme-linked immunosorbent assay; HDL, High density lipoprotein; HMW, High molecular weight; IP, Immuno-precipitation; NAD, Non-AD or non-dementia control; ND, Not detected; PAGE, Polyacrylamide gel electrophoresis; SDS, Sodium dodecyl sulfate; SEC, Size exclusion chromatography; WB, Western blot.

#### Method of detection

ApoE/Aβ complex is defined by the method of detection, particularly the stringency of the assay conditions and the method used to isolate the endogenous complex. Methods utilized to measure apoE/Aβ complex, in order of decreasing stringency, include: gel-shift assay of SDS-PAGE (under reducing or non-reducing conditions), with Western blot analysis (WB) [33,42,59,66,67]; density gradient ultracentrifugation [68]; non-denaturing gradient gel electrophoresis [59]; co-immunoprecipitation (IP) [46]; size exclusion chromatography (SEC)/gel-filtration; and solid-phase binding assays [29,38,45,47,51,69]. Using primarily SDS-PAGE or SEC-isolation followed by dot blot (DB) or WB (Table 1), apoE/Aβ complex is detected when exogenous Aβ is combined with apoE-containing lipoproteins from human plasma, CSF and cell culture supernatants. However, methods with relatively high stringency can result in disruption of the apoE/Aβ complex, thus confounding interpretation of the data. The influence of assay stringency is highlighted by the effect of detergent during SDS-PAGE with WB analysis; exogenously produced SDS-stable apoE3/Aβ levels are greater than apoE4/Aβ [33], but comparable when analyzed by non-denaturing gel electrophoresis [59]. These data are consistent with an SDS-stable apoE3/Aβ complex, and an apoE4/Aβ complex that is disrupted by SDS (for review [56]). As well, the inclusion of a reducing agent during SDS-PAGE (β-mercaptoethanol, β-ME; dithiothreitol, DTT) disrupts both the SDS-stable apoE3/Aβ and apoE4/Aβ complexes [30,31,66].

For endogenously produced apoE/Aβ complex (Table 2), assay stringency also affects the levels of apoE/Aβ complex. Endogenous apoE/Aβ complex has been detected in the soluble fraction of human brain [46] and cerebrospinal fluid (CSF) [53,63] by SEC isolation followed by DB or WB. The potential for endogenous apoE/Aβ complex to be disrupted by the method of detection was demonstrated in one of the earliest apoE/Aβ complex studies by Russo and co-workers [46]. When analyzed by co-IP, soluble brain apoE/Aβ complex levels were lower in AD compared to non-AD (NAD) patients. Importantly, complex from AD patients was less stable to SDS-PAGE with WB analysis and more susceptible to enzymatic degradation [46].

#### Source of apoE/Aβ complex

ApoE is the major apolipoprotein present on CNS lipoproteins (for review [70]). In the CNS, apoE is produced primarily by glia (astrocytes and microglia), although under certain conditions neuronal apoE expression may occur [71]. Because apoE is an apolipoprotein, interactions with Aβ depend on the lipidation state of the apoE-containing lipoproteins (see below) i.e. whether the apoE is purified [33,42,51,66], lipid-poor [33,51,54], reconstituted with lipids from HDL [51], astrocyte-secreted [59], CSF-derived [29], or isolated plasma-lipoproteins [42,66,68]. Thus, evaluating the exogenous interactions between apoE and Aβ influenced by the source of apoE. For example, purified apoE4 binds Aβ with a higher affinity than apoE3 [30,66]. However, this affinity is reversed using lipidated apoE; levels of apoE3/Aβ complex are significantly greater than apoE4/Aβ complex [30,33,66].

#### Interpretation

Landmark studies are consistent with the decreased stability of exogenous apoE4/Aβ complex compared to the apoE3/Aβ when a "physiological relevant" source of apoE is used. Further, the presence of both detergent and a reducing agent significantly reduce complex levels, with apoE4/Aβ affected more than apoE3/Aβ. Importantly, soluble levels of endogenous apoE/Aβ complex levels are lower and the complex less stable in AD versus NAD brain samples, suggesting a link between apoE isoform-specific formation of apoE/Aβ complex and AD, for which *APOE4* is the greatest risk factor.

### Recent data

#### Is there a soluble apoE/Aβ complex?

A recent publication by Verghese and co-workers brought into question the significance of the apoE/Aβ complex for modulating Aβ levels [65]. Although the overall goal of the study was to determine the effect of apoE/Aβ complex on Aβ metabolism, the authors noted that apoE forms minimal complex with Aβ [65]. For exogenously produced apoE/Aβ complex, only 5% of cell-derived Aβ formed a complex with apoE (astrocyte derived or lipid reconstituted) when analyzed using gradient ultracentrifugation, SEC followed by ELISA analysis and fluorescence correlation spectroscopy (FCS) [65]. The apparent difference between these results and previous studies, where cell-derived apoE and Aβ formed a significant amount of apoE/Aβ complex, was attributed to a non-physiologically high ratio of Aβ to apoE. Indeed, previous studies often added Aβ in excess of apoE (Table 1), whereas Verghese and co-workers used "physiological" ratios of apoE to Aβ [65]. However, in CSF, plasma and brain homogenates from humans and FAD-Tg mice, the concentrations of Aβ are significantly lower than apoE. For example, the apoE:Aβ ratios reported for human CSF are in the range of 1:0.006-0.02 [72-74]. In addition, density gradient centrifugation is known to induce loss of (apolipo)proteins from lipoproteins [67], which would likely effect the components of the apoE/Aβ complex isolated by this method. Finally, FCS analysis requires β-ME, which disrupts apoE/Aβ complex [42].

Verghese and co-workers report similar results for endogenous apoE/Aβ complex in human CSF, with only ~5% of apoE and Aβ co-eluting from SEC in fractions analyzed by ELISA [65]. This is in contrast to a previous study that demonstrated ~100% co-elution of apoE and Aβ from human CSF [63]. Although no data are presented, the hypothesis is that concentration of the CSF 10-30-fold prior to SEC as used in the previous publications [63,75], may have induced apoE/Aβ complex formation.

#### APOE modulates soluble apoE/Aβ levels measured by ELISA

To determine the effect of apoE isoform on apoE/Aβ complex levels under non-stringent conditions, we developed an apoE/Aβ complex ELISA to detect both the total levels of apoE/Aβ complex and, after the addition of SDS, the stability of the apoE/Aβ complex [4]. With this technique, using cell-derived apoE and synthetic Aβ42 at physiological ratios (exogenous complex), total apoE/Aβ complex levels were equivalent for the three apoE isoforms, whereas SDS-stability of the apoE/Aβ complex was isoform-specific; apoE2/Aβ > apoE3/Aβ > apoE4/Aβ, consistent with previous results utilizing alternative, non-stringent detection methods [33,41,42,54]. In addition, using this ELISA, the apoE/Aβ complex is less stable at a low pH [4], also in agreement with previous data [66].

With the apoE/Aβ complex ELISA, we also identified an *APOE* genotype-specific difference in endogenous apoE/Aβ complex levels in EFAD-Tg mice and human samples [4]. In EFAD-Tg mice, soluble levels of apoE4/Aβ complex were lower and less stable compared to apoE3/Aβ and apoE2/Aβ. Further, soluble oAβ levels were higher in E4FAD mice compared to E2FAD and E3FAD mice, suggesting that apoE/Aβ complex may modulate oAβ levels. In human synaptosome preparations and CSF, apoE/Aβ levels were lower in AD compared to NAD samples, and with *APOE4* compared to *APOE3* in the AD cohort. Importantly, in human CSF, oAβ increased and was greater with *APOE4* in the AD cohort, in contrast to total Aβ42 levels that decrease with AD compared to NAD, with levels at the limit of detection in the AD cohort with both *APOE3* and *APOE4*. Taken together, the low levels of soluble apoE4/Aβ complex and high levels of soluble oAβ suggest a potential basis for *APOE4*-induced AD risk.

### Interpretation

As we seek to define the role of apoE/Aβ complex in the parenchyma of the brain, extracting intact lipoproteins from tissue homogenates is critically important but procedurally problematic. For now, the optimal CNS samples are soluble brain homogenates or CSF. In addition, as discussed in the previous section, multiple factors are known to influence the detection of apoE/Aβ complex levels. One technique is not necessarily superior to another; each has merits and limitations. Thus, future studies may utilize multiple, complementary techniques for sample analysis. Overall, based on the literature and our recent data demonstrating an inverse relationship between the levels of soluble apoE/Aβ complex and oAβ, we hypothesize that complex plays a significant role in modulating oAβ levels. The lower levels and instability of apoE4/Aβ complex compared to apoE3/Aβ suggests a potential mechanism for the APOE4-induced risk for AD.

## Does apoE isoform-specific lipoprotein lipidation affect apoE/Aβ complex levels?

CNS apolipoproteins are exclusively produced within the brain and apoE is the major apolipoprotein in the CNS [70]. The biogenesis of CNS apolipoproteins occurs primarily in the interstitial fluid and a proposed model of lipoprotein remodeling includes: 1) glial cells secrete nascent apoE-containing lipoproteins that are lipid-poor and discoidal in shape; 2) ABCA1 and ABCG1 in glia and neurons efflux free cholesterol to these lipoproteins, increasing the lipidation state of this now intermediate lipoprotein particle; and 3) intermediate particles mature and likely become spherical through acquiring a cholesteryl ester core and additional phospholipids [76]. Thus, apoE isoform-specific lipoprotein lipidation is a critical parameter that modulates the overall function of these lipoproteins, including the formation of apoE/Aβ complex.

### ApoE modulation of lipoprotein lipidation

The general dogma in the field is that apoE4 in the CNS is less lipidated than apoE3. However, it is difficult to isolate and analyze intact CNS lipoproteins, thus limiting direct study of the lipidation state of apoE4-particles versus apoE3-particles. However, the functional consequences of conditions predicted to affect the lipidation state of lipoproteins have provided valuable insights. For example, *in vitro* data demonstrate that glia-mediated degradation of apoE is increased and cholesterol release is reduced in primary glial cultures expressing apoE4 compared to apoE3 [77,78]. *In vivo* study of Tg mice using over-expression or knock-out of the ABC transporters has also provided important information on the functional effects of the lipidation state of CNS lipoproteins [79-82]. For example, Fitz and co-workers demonstrated that introducing ABCA1^-/+^ to an FAD/apoE-Tg mouse results in increased plaque deposition and a general AD phenotype in mice expressing apoE4 but not apoE3 [82]. In addition, we recently developed a three-step sequential protein extraction protocol (TBS, TBSX, formic acid/FA). A non-ionic detergent Triton X-100 (TBSX) is used to in an attempt to release apoE from lipoprotein particles without inducing the formation of new micelles, as can occur with ionic detergents such as SDS [12,83]. Using EFAD mouse brain extraction fractions, we demonstrated that while total brain apoE4 levels are lower compared to apoE2 and apoE3*,* this decrease is seen only in the TBSX fraction [12]. These data provide evidence that less apoE4 is associated with lipoproteins and thus apoE4 may be less lipidated than apoE2 and apoE3.

#### ApoE lipoprotein lipidation and apoE/Aβ complex levels

Biophysical data demonstrates that, compared to apoE3, apoE4 has an increased propensity to populate an intermediate molten globule state during denaturation, suggesting a less stable conformation [84-86]. Thus, the decreased stability of apoE4-containing lipoproteins may, in part, explain the decreased stability of the apoE4/Aβ complex compared to the apoE3/Aβ complex. An additional consideration is whether apoE and Aβ form a direct complex, or whether Aβ interacts with the lipid surface of apoE-containing lipoproteins, which likely comprise a relatively larger surface area of the lipoprotein particle compared to apoE. Simplistically, if apoE4-containing lipoproteins are less lipidated than apoE3-containing lipoproteins, then a smaller surface area is provided on a less stable lipoprotein for interactions with Aβ.

### Interpretation

Identifying whether apoE4-containing lipoproteins are less lipidated than apoE3- containing lipoproteins, and which lipids differ between the two, are important considerations. If, as hypothesized, apoE4-containing lipoproteins are less lipidated than apoE3 containing lipoproteins, this may explain the reduced levels and stability of the apoE4/Aβ complex.

## How might apoE/Aβ complex modulate soluble Aβ levels?

The functional key question in this field is how apoE/Aβ complex modulates soluble Aβ levels. Although there are a number of proposed mechanisms, they generally fall into 2 categories: 1) modulation of Aβ clearance, and 2) Aβ aggregation.

### Aβ clearance

The Aβ clearance rate is slower with apoE4 compared to apoE3 in PDAPP/apoE-TR mice [87] and in apoE-TR mice after a bolus brain injection of Aβ [88]. ApoE exhibits an isoform-specific effect on a number of cellular processes that modulate soluble Aβ clearance, and the mechanisms underlying these effects may be influenced by apoE/Aβ complex levels. Compared to apoE3, apoE4 results in a decrease in Aβ levels via: 1) clearance via glia [89-93], neurons [94-96], and the blood-brain barrier [88,97]; 2) intracellular and parenchymal enzymatic degradation [93]; and 3) drainage via the interstitial fluid (ISF) [87] and perivasculature [98]. One potential mechanism is that soluble apoE3/Aβ complex may reduce soluble Aβ levels via clearance, whereas the lower levels of soluble apoE4/Aβ complex result in higher soluble Aβ levels, particularly oAβ. In terms of cellular clearance, the literature appears contradictory, likely due to the variety of models and reagents used, including; glia versus neurons, mouse versus human cells, mouse versus human apoE, source of apoE (purified protein or lipidated particle), source and aggregation state of the Aβ, etc. In a particularly relevant study, Nielson and co-workers used primary human astrocytes to address the role of the human apoE isoforms in binding and uptake of Aβ42. Their results demonstrated that both apoE3 and apoE4 inhibited the uptake of oAβ42 but not fibrillar forms of the peptide [99].

### Aβ aggregation

Data are conflicting as to the effect of apoE/Aβ complex on Aβ aggregation. Previous publications have demonstrated that apoE promotes Aβ aggregation, resulting in retention of the peptide; within glia [99], as intraneuronal Aβ [100], or in the parenchyma as either diffuse Aβ deposits or amyloid [12]. *APOE4* is associated with an increase in amyloid deposition, suggesting that apoE functions as a "pathological chaperone", an effect that might actually be beneficial [101]. However, as demonstrated *in vitro*, apoE isolated from human brain increases the oligomerization of Aβ [27], an effect that is greater with apoE4 compared to apoE3. This is consistent with the hypothesis that apoE binds to oAβ and prevents further aggregation [64]. These results would favor the view that lower levels of apoE/Aβ complex are beneficial [102,103].

#### Interpretation

Delineating the effect of apoE/Aβ complex on Aβ clearance and aggregation is complex due to the multiple mechanisms involved in these processes. It is particularly difficult to interpret *in vitro* data as the studies focus on multiple, different intercellular and intracellular pathways. At this point, research appears to support a role for apoE/Aβ complex in both promoting Aβ clearance and Aβ aggregation, both as insoluble amyloid and soluble oAβ.

## How can the apoE/Aβ complex be targeted therapeutically?

Therapeutics that target the apoE/Aβ complex can be broadly divided in those that increase the ability of apoE-containing lipoproteins to form an apoE/Aβ complex and those that disrupt apoE/Aβ complex.

### Therapies to increase apoE/Aβ complex

#### Nuclear receptor agonists

Increasing the lipidation of apoE-containing lipoproteins may increase apoE/Aβ complex levels and decrease oAβ levels. As described above, the transporters ABCA1 and ABCG1 expressed by glia and neurons in the CNS are considered the major transporters of lipid to the nascent CNS apoE-containing lipoproteins [76]. Agonists for the nuclear receptors PPARγ [104-106], LXR (reviewed in [107]) and RXR [108-111] increase ABCA1 and ABCG1 expression and decrease soluble Aβ levels in FAD-Tg mice. However, controversy exists over the activity and mechanism of action of these agents, highlighted by recent data with the RXR agonist bexarotene [112]. Initial data demonstrated that bexarotene increased apoE levels and decreased soluble Aβ within hours and significantly reduced insoluble Aβ after three days [112]. These data were purported to be consistent with an apoE-induced increase clearance of both soluble and insoluble Aβ. However, very recent findings indicate that bexarotene treatment of FAD-Tg mice reduced soluble Aβ and improved memory without changing amyloid or apoE levels but with increased ABCA1 expression [108-111]. Therefore, a potential novel mechanism of action of RXR agonists is an increase in ABCA1 expression, which results in increased lipid content/lipidation of apoE-containing lipoproteins, a process that may be particularly beneficial to lipid-poor apoE4-containing lipoproteins. This increased lipidation likely results in an increase in apoE/Aβ complex levels and a decrease in soluble Aβ42 levels, as discussed in Section 4 above.

#### Dietary strategies

A recent publication highlighted the effect of *APOE* and diet on Aβ levels and apoE lipidation in patients with MCI [113]. The overall conclusion was that with a high saturated fat or glycemic diet, apoE was less lipidated and it formed lower levels of apoE/Aβ complex, resulting in reduced Aβ clearance and increased Aβ oligomerization and toxicity [113]. Thus, dietary interventions may prove effective for raising apoE/Aβ complex levels.

### Therapies to block apoE/Aβ complex

#### Aβ12-28P

The potential for apoE to increase Aβ aggregation, and even specifically oligomerization, led to the development of the Aβ12-28P, an Aβ peptide purported to block the formation of apoE/Aβ complex [102,103]. *In vivo* data demonstrate that Aβ12-28P lowers insoluble Aβ both in the brain [102,103] and vasculature [114] of FAD-Tg mice. However, data on the effect of this peptide on apoE/Aβ complex levels are limited. In addition, Aβ12-28P has not been tested in the absence of apoE (apoE-KO mice), leaving open the possibility that it may be interacting directly with Aβ to reduce aggregation. Therefore, the mechanism of action of Aβ12-28P *in vivo* is currently unclear.

#### Interpretation

A major hurdle to interpreting how therapeutic or dietary interventions modulate apoE/Aβ complex, Aβ accumulation, and ultimately AD, is their pleiotropic mechanisms of action. For example, LRX/RXR/PPARγ agonists display anti-inflammatory actions, which may or may not be dependent on lipoprotein lipidation, and ABCA1 may directly clear Aβ. However, data with nuclear receptor agonists suggests that increasing ABCA1/ABCG1 expression will increase lipoprotein lipidation, apoE/Aβ complex levels and Aβ clearance, thus reducing soluble oAβ levels.

## Conclusions

Our hypothesis is that the *APOE4*-induced risk of AD is consistent with the following pathway to neurodegeneration: Compared to apoE3, apoE4-containing lipoproteins are less lipidated, which reduces stability, resulting in lower levels the apoE4/Aβ complex. Reduced levels of apoE4/Aβ complex result in increased Aβ accumulation, specifically oAβ levels. Thus, increasing the lipidation of apoE-containing lipoproteins may reduce Aβ accumulation, an effect particularly effective with the lipid-poor apoE4-lipoproteins.

## Abbreviations

ABC: ATP-binding cassette transporter; Aβ: Amyloid-β; AD: Alzheimer’s disease; apoE: Apolipoprotein E; apoE-TR: ApoE targeted replacement mice; β-ME: β-mercaptoethanol; CNS: Central nervous system; CSF: Cerebrospinal fluid; DB: Dot blot; DTT: Dithiothreitol; EFAD mice: 5xFAD mice crossed with apoE-TR mice; FAD: Familial Alzheimer’s disease; FAD-Tg: Transgenic mice expressing APP and/or PS1 with FAD mutations; FCS: Fluorescence correlation spectroscopy; IHC: Immunohistochemistry; IP: Immunoprecipitation; ISF: Interstitial fluid; NAD: Non-AD; oAβ42: Oligomeric Aβ; SDS-PAGE: Sodium dodecyl sulphate polyacrylamide gel electrophoresis; Tg: Transgenic; WB: Western blot; 5xFAD: Mice containing 5 FAD mutations.

## Competing interests

In collaboration with Skip Binder, MJL developed the antibody MOAB-2 used to develop the apoE/Aβ complex and oAβ ELISAs. MOAB-2 is licensed as a research tool to multiple companies. Also for research purposes, Biosensis Pty Ltd has licensed the rights to ELISA kits with MOAB-2 for oAβ and apoE/Aβ.

## Authors’ contributions

LMT: Prepared the manuscript and Tables. SM: Provided input on, and preparation of, the Tables with LMT and MJL. VS: Provided significant input on final manuscript preparation, revision and submission. SE/GWR/GB: Collaborators who advised and provided significant input on the content and direction of the manuscript text. MJL: Provided fundamental contributions to the manuscript conception and preparation. All authors read and approved the final manuscript.
